# Tailor-made solvents for microbial carotenoids recovery

**DOI:** 10.1007/s00253-024-13049-x

**Published:** 2024-02-24

**Authors:** Cassamo U. Mussagy, Henua U. Hucke, Nataly F. Ramos, Helena F. Ribeiro, Mariana B. Alves, Ahmad Mustafa, Jorge F. B. Pereira, Fabiane O. Farias

**Affiliations:** 1 Escuela de Agronomía, Facultad de Ciencias Agronómicas y de los Alimentos, Pontificia Universidad Católica de Valparaíso, 2260000 Quillota, Chile; 2 CIEPQPF, Department of Chemical Engineering, Faculty of Sciences and Technology, University of Coimbra, Rua Sílvio Lima, Pólo II—Pinhal de Marrocos, 3030-790 Coimbra, Portugal; 3 Faculty of Engineering, October University for Modern Sciences and Arts (MSA), Giza, Egypt; 4 Department of Chemical Engineering, Polytechnique Center, Federal University of Paraná, Curitiba, PR Brazil

**Keywords:** Microorganisms, Carotenoids, Ionic liquids, Deep eutectic solvents, Alternative solvents

## Abstract

**Abstract:**

In recent years, microbial carotenoids have emerged as a promising alternative for the pharmaceutical and food industries, particularly in promoting human health due to their potent antioxidant and antimicrobial properties. Microbial carotenoids, particularly those produced by yeast, bacteria, and microalgae, are synthesized intracellularly, requiring the use of solvents for their effective extraction and recovery. The conventional use of toxic volatile organic solvents (VOCs) like hexane, petroleum ether, and dimethyl sulfoxide in the extraction of microbial carotenoids has been common. However, ongoing research is introducing innovative, non-toxic, environmentally friendly tailor-made solvents, such as ionic liquids (IL) and deep eutectic solvents (DES), indicating a new era of cleaner and biocompatible technologies. This review aims to highlight recent advancements in utilizing IL and DES for obtaining carotenoids from microorganisms. Additionally, we explore the utilization of in silico tools designed to determine the solubilities of microbial carotenoids in tailor-made DES and ILs. This presents a promising alternative for the scientific community, potentially reducing the need for extensive experimental screening of solvents for the recovery of microbial carotenoids in the separation processing. According to our expert perspective, both IL and DES exhibit a plethora of exceptional attributes for the recovery of microbial carotenoids. Nevertheless, the current employment of these solvents for recovery of carotenoids is restricted to scientific exploration, as their feasibility for practical application in industrial settings has yet to be conclusively demonstrated.

**Key points:**

• *ILs and DES share many tailoring properties for the recovery of microbial carotenoids*

• *The use of ILs and DES for microbial carotenoid extraction remains driven by scientific curiosity.*

• *The economic feasibility of ILs and DES is yet to be demonstrated in industrial applications.*

## Introduction

Natural pigments, or biopigments, are gaining popularity as alternatives to synthetic pigments (Mussagy et al. 2023; Xing et al. 2023). This shift is driven to increasing consumer concerns about chemophobia and the growing demand for eco-friendly, biocompatible, and safe compounds (Meléndez-Martínez et al. 2019; Mapelli-Brahm et al. 2020). For instance, several biopigments such as carotenoids, anthocyanins, among others are essential components of a health-preserving diet. Carotenoids, a fat-soluble group of pigments responsible for yellow, orange, and red colors, are naturally found in algae, fungi, bacteria, and numerous vegetables (Mussagy et al. 2019a; Meléndez-Martínez 2019; Liu et al. 2020). These natural pigments are classified into two main categories: carotenes, such as phytoene, lycopene, and β-carotene; and xanthophylls, including astaxanthin, zeaxanthin, cantaxanthin, among others (Mussagy et al. 2019a). Their prominence extends to important roles in the food, feed, cosmetic, nutraceutical, and pharmaceutical industries. Recognized as health-promoting compounds, carotenoids are widely incorporated into our daily diet, contributing to the reduction of several diseases including neural disorders, some types of cancers, cardiovascular diseases, cataracts, acute lung injury, among others (Meléndez-Martínez 2019).

In nature, plants are the primary source of natural carotenoids, but microbial sources, including bacteria, yeast, and microalgae, are becoming more popular for these pigments. Microorganisms produce carotenoids as a defense against photo-oxidative damage, helping them thrive in light- and air-rich environments (Toti et al. 2018; Genç et al. 2020). The carotenoid content in microbial sources varies depending on factors such as the specific microorganism, species, strain, and cultivation conditions. In fact, the use of microbial sources for natural carotenoids offers practical advantages, including rapid growth, efficiency, adaptability, year-round availability, and reduced environmental impact. These benefits have led to the growth of the microbial carotenoids market, which was estimated to increase from 1.5 billion USD in 2019 to 2.0 billion USD by 2026 (Markets and Markets. 2023). The growth of this market is primarily driven by the extraction of safe microbial carotenoids like astaxanthin, lutein, and β-carotene, which serve as effective substitutes to chemically derived carotenoids.

Carotenoids are produced intracellularly in several microorganisms (Mussagy and Dufossé 2023). Consequently, apart from their high production levels, the primary issues related to employing microorganisms for carotenoid production involve the necessity of implementing additional extraction processes for the recovery of these intracellular pigments. To date, there is no universally recognized method for extracting carotenoids from microbial biomasses. The efficiency of recovery depends on several factors, including carotenoid polarity, biomass type, microorganism nature, solvent employed, moisture content, and cell wall composition, among others (Singh et al. 2015; Hernández-Almanza et al. 2017; Martínez et al. 2020). Traditionally, the recovery of more hydrophobic carotenoids, such as phytoene, lycopene, and β-carotene, is favored by employing non-polar volatile organic compounds (VOCs) like petroleum ether, hexane, and ethyl acetate as extractants. In contrast, slightly polar VOCs such as dimethyl sulfoxide, ethanol, methanol, acetone, among others are commonly used to extract astaxanthin, zeaxanthin, and cantaxanthin (Singh et al. 2015; Hernández-Almanza et al. 2017; Martínez et al. 2020).

Briefly, the recovery of intracellular microbial carotenoids begins with biomass pretreatment, either wet or dry. This is succeeded by cellular disruption or cell-wall permeabilization, often achieved through mechanical techniques. Carotenoid solubilization is then carried out using VOCs, and further purification is employed to remove unwanted compounds (i.e., proteins, sugars, and lipids) (Mussagy et al. 2020). To date, conventional methods, along with ‘advanced’ techniques such as microwave-assisted extraction (MAE), ultrasound-assisted extraction (UAE), supercritical fluid extraction (SFE), and pressurized liquid extraction (PLE), can be employed for carotenoids recovery. These approaches may also involve the use of volatile organic compounds (VOCs) (Singh et al. 2015). However, the use of non-environmentally friendly VOCs such as hexane, acetone, dimethyl sulfoxide, dichloromethane, and other, either individually or in combination, for the recovery of microbial carotenoids can pose environmental and health concerns including air pollution, greenhouse gas (GHG) emissions, human health risks, and environmental contamination (Mussagy et al. 2022b).

Hence, the pursuit of eco-friendly solvents based on green chemistry principles is of paramount importance. Moreover, tailor-made solvents such as ionic liquids (ILs) and deep eutectic solvents (DES) may provide higher recovery yields of microbial carotenoids, as well as allowing enhanced selectivity, higher solvation properties, and good recyclability performance (mainly for ILs), in contrast to conventional solvents (Vanda et al. 2018; Binnemans and Jones 2023). ILs are tailor-made salts composed of organic cations and anions, typically with melting points < 100 °C, possess low volatility and non-flammability, and exhibit high stability when exposed to harsh thermal and chemical conditions (Ventura et al. 2017). DES is a new type of tailor-made solvent composed of mixtures of two or more compounds with a melting point below the melting point of the individual components. DES are formed by mixing a hydrogen bond acceptor (HBA) and a hydrogen bond donor (HBD) (Vanda et al. 2018; Hansen et al. 2021;  Ueda et al. 2023). ILs and DES can be synthesized using selected starting materials and a broad array of combinations, enabling the creation of numerous solvents with specific physical properties and the ability to solubilize diverse carotenoids. The starting materials of these tailor-made solvents are often abundant in nature and are generally regarded as non-toxic, biocompatible, and biodegradable.

**Fig. 1:**
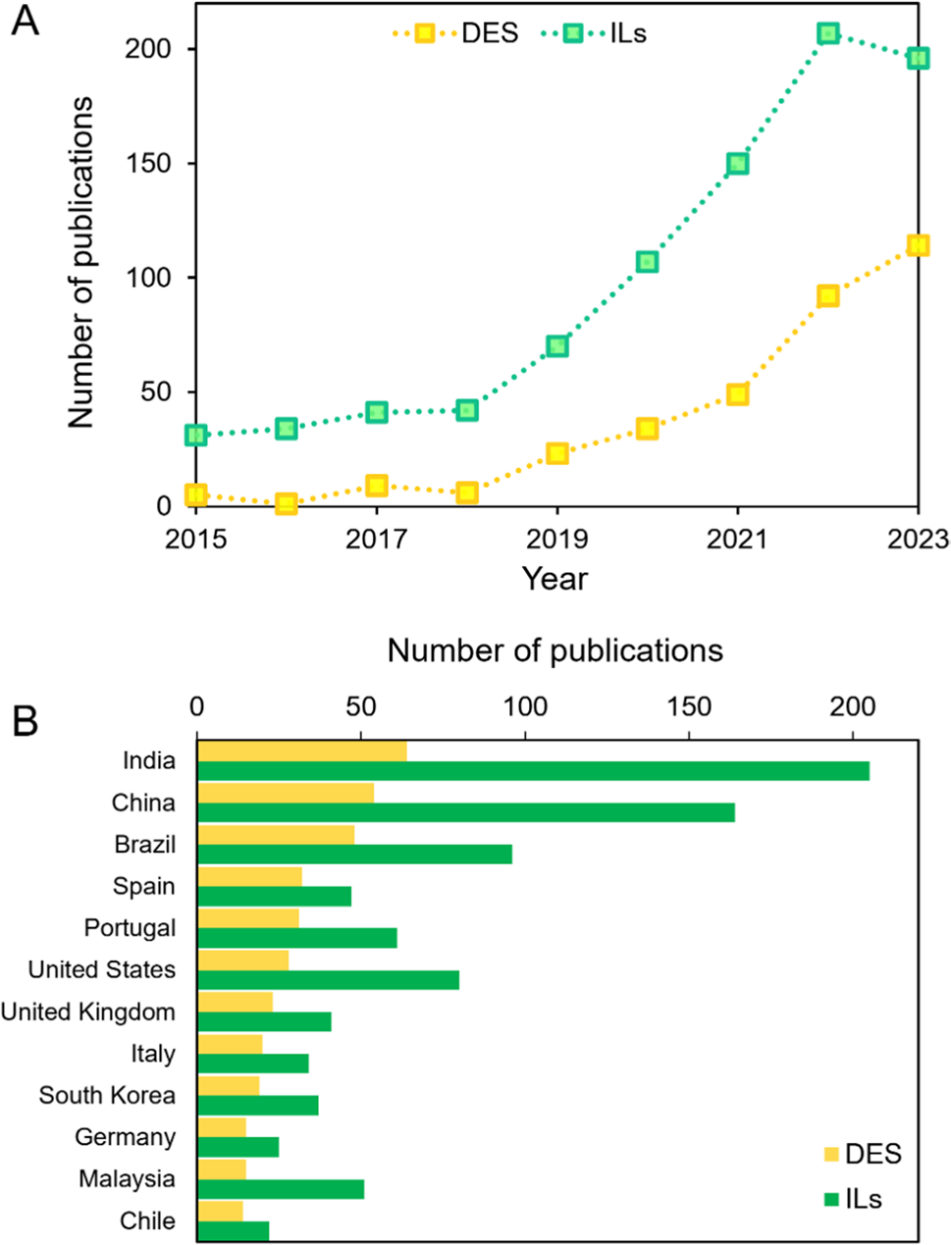
**A** ILs and DES cumulative number of publications *per* year (from 2015 to 2023); **B** contributions of most significant countries of papers published in the same period (Scopus database)

In the last 10 years, the interest in tailor-made solvent for extraction of microbial carotenoids has grown due to their numerous advantages. This trend is evident from the bibliometric analysis we conducted on the Scopus® database using the search terms [‘(ALL (“carotenoids”) AND “microorganisms” AND “eutectic solvents” AND “yeast” OR “bacteria” OR “microalgae” AND PUBYEAR > 2014 AND PUBYEAR < 2023)’] for eutectic solvents and [‘ALL (“carotenoids”) AND “microorganisms” AND “ionic liquids” AND “yeast” OR “bacteria” OR “microalgae” AND PUBYEAR > 2014 AND PUBYEAR < 2023’] for ionic liquids (Fig. 1). From 2014 to 2023, over 1211 documents were published on the recovery of microbial carotenoids using DES or ILs, with approximately 72% related to ILs and 28% to DES (Fig. 1A). These results indicate the growing interest of the scientific community in the use of more biocompatible solvents, with publications increasing from 5 (DES) and 31 (ILs) in 2015 to nearly 114 (DES) and 196 (ILs) in 2023. The analysis also reveals that India, China, and Brazil are the countries with the largest number of documents published from 2015 to 2023, which aligns with the increasing demand for more natural products in these countries (Fig. 1B).

The use of tailor-made solvents in research holds great promise, serving not only as an academic and scientific instrument but also as a crucial component in the development of novel bioprocesses, ranging from pilot to industrial scales. The success of each procedure depends on the chosen technique, tailor-made solvents consisting of cations, anions, or HBA/HBD families, and the unique characteristics of microbial biomass. The remarkable biological attributes of microbial carotenoids have ignited heightened interest across various industries, resulting in a search for sustainable technologies that can enhance the extraction of these carotenoids from within microbial systems. While previous reviews have examined the efficiency of conventional VOCs in extraction processes, there are still unexplored aspects, such as the impact of ILs and the nature of DES on the recovery and purification of microbial carotenoids, as well as the integration of extraction and purification processes to optimize purities while minimizing losses.

In light of these considerations, this review explores the use of tailor-made solvents, particularly DES and ILs, for the recovery of carotenoids from microbial sources. Additionally, we investigate in silico tools that can predict the solubility of microbial carotenoids in tailor-made solvents, offering readers valuable information to make informed choices that minimize unnecessary resource usage.

## In silico tools for tailoring solvents

Predictive models are valuable tools for screening the optimal solvents for biomolecules recovery, as they reduce the need for time-consuming and resource-intensive experimental testing. In the context of tailoring-made solvents, their importance becomes even more evident, given the vast array of precursors and combinations that can be employed to prepare DES or synthesize ILs. The COSMO (COnductor-like Screening MOdel) models exemplify in silico tools that utilize computational methods for predicting electrostatic interactions between molecules and solvents. Initially introduced by Klamt and Schüürmann (1993) (Klamt 1995) as the COSMO-RS (COnductor-like Screening MOdel for Real Solvents), this model paved the way for subsequent advancements. Building upon COSMO-RS, Lin and Sandler (2002) further developed another approach, leading to the creation of COSMO-SAC (COSMO Segment Activity Coefficient) Model. These models are based on evaluating the charge distribution in the molecules, which can be used to explain the differences in polarity through the sigma-profile (σ-profile) (Oliveira et al. 2020). The σ-profile is unique to each compound and illustrates the distribution of the screening surface density of the molecules. Therefore, it is possible to compare the σ-profiles of the solute and the solvents (or their precursors) to select the most suitable solvent for solute solubilization. Furthermore, COSMO-based models enable the determination of a solvent’s capacity to solubilize the target solute through the calculation of the activity coefficient at infinite dilution (${ln\gamma }_{i}^{\infty }$) calculation (Gerber and Soares 2010). A lower value of the ${ln\gamma }_{i}^{\infty }$ indicates a higher affinity between the solute and the solvent.

**Fig. 2:**
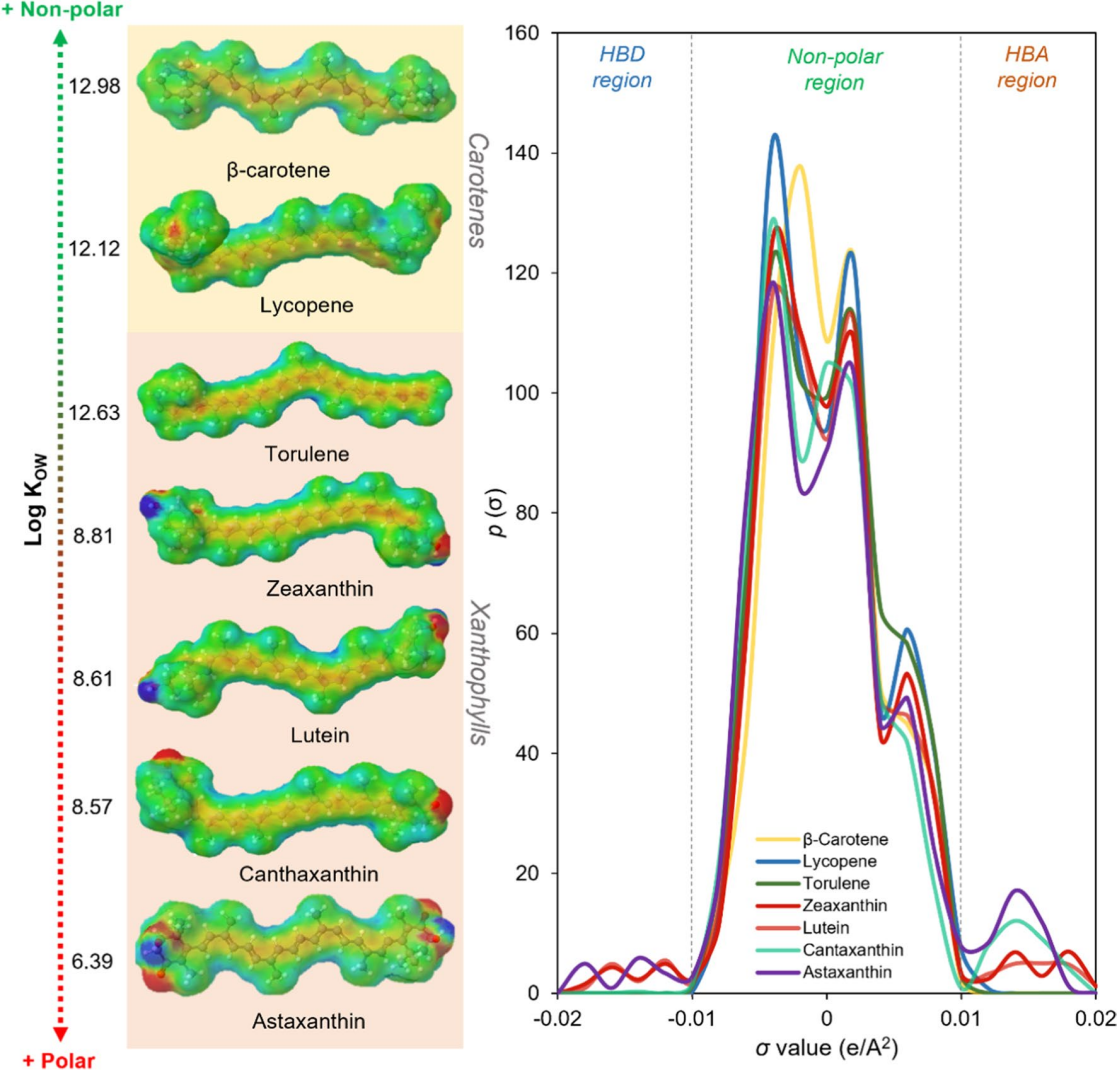
Sigma-profile and charges distribution of some specific carotenoids. Note: Log K_OW_ were predicted using COSMO-SAC model

To represent the applicability of these computational models, the σ-profile of some carotenoids was obtained by the COSMO-SAC model (Gerber and Soares 2010; Ferrarini et al. 2018) (as depicted in Fig. 2). The σ-profile can be divided into three regions: the non-polar region of the molecule, which is situated between − 0.01 and + 0.01 e/Å^2^; while the regions lower than − 0.01 e/Å^2^ and higher than + 0.01 e/Å^2^ are considered hydrogen bond acceptor (HBA) and hydrogen bond donor (HBD) regions, respectively. The charge distribution can also be easily comprehended by the colors around the molecules, *i.e.*, green areas are the non-polar region, and the red and blue colors correspond to the positive and negative polarization charges, respectively. Figure 2 shows that carotenes, which lack hydroxyl groups in their structure, exhibit only non-polar regions. Consequently, solute–solvent interactions will exclusively involve covalent bonds, making strong non-polar solvents preferable. On the other hand, xanthophylls possess hydroxyl groups, which introduce polar regions. In this case, it is necessary to consider weak non-polar solvents along with some polar regions for optimal solute–solvent interactions. This can be reinforced by the logarithm of the octanol–water partition (Log K_OW_) of the carotenoids, also presented in Fig. 2. The higher is this parameter, the more hydrophobic the target compound is.

**Fig. 3:**
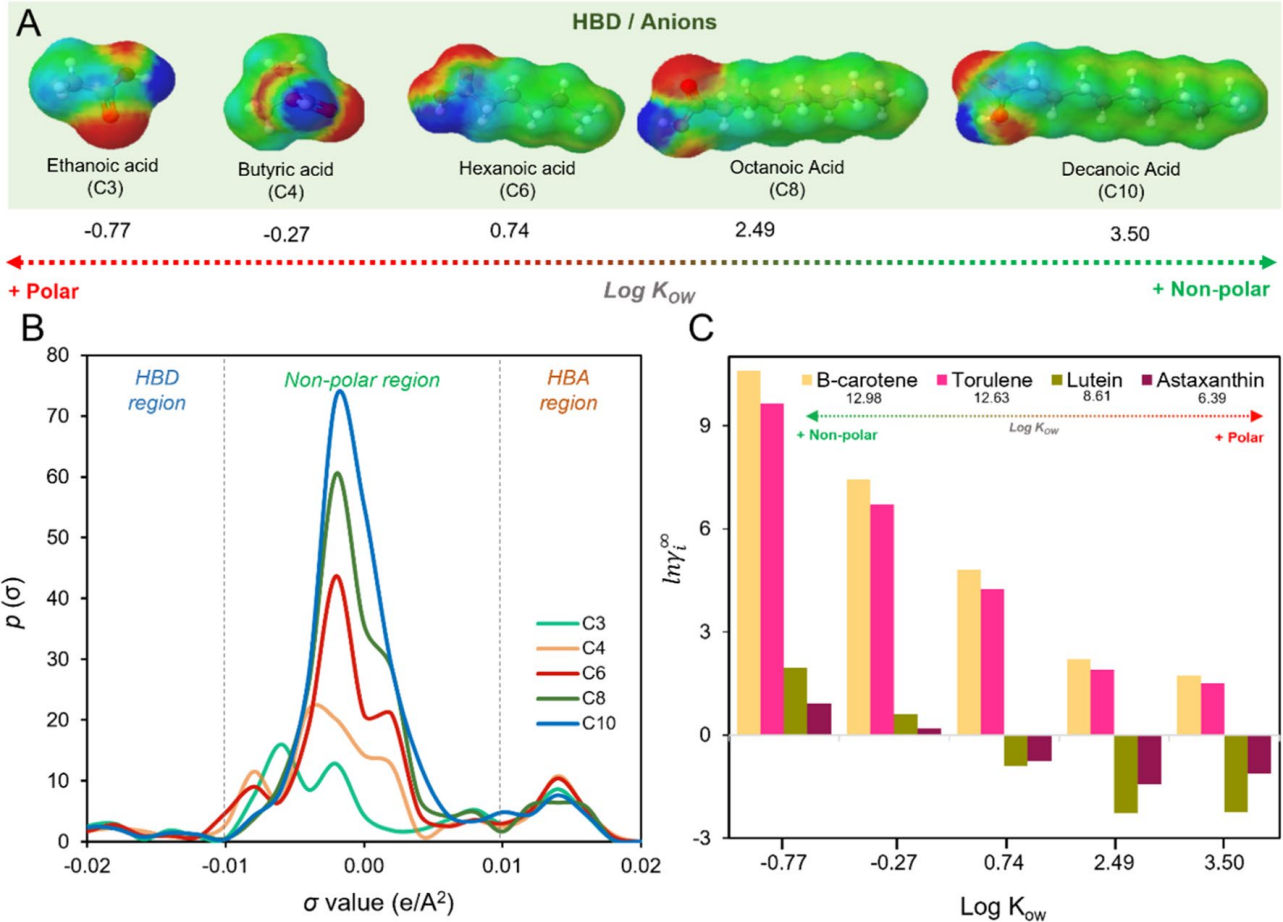
**A** Charges distribution and **B** Sigma-profile of some commonly used as DES and ILs hydrogen bond donor and anions precursors, respectively. **C** Correlation between Log K_OW_ and ${ln\gamma }_{i}^{\infty }$ predicted using COSMO-SAC model

After understanding the carotenoid sigma-profile, it is important to predict the same parameter for solvents to ensure effective recovery. Following the same approach, Fig. 3 presents the sigma-profile of carboxylic acids, such as ethanoic, butyric, hexanoic, octanoic, decanoic acids, which are commonly used as DES and ILs hydrogen bond donor (HBD) and anion precursors, respectively. To gain a deeper understanding of the importance of carotenoids-solvent interactions, the ${ln\gamma }_{i}^{\infty }$ calculation was performed for four carotenoids, as shown in Fig. 3. As expected, the longer is the carbon chain size of the carboxylic acids, the greater is the non-polar area of these compounds on the σ-profile (Fig. 3B), leading to increased similarity with carotenoids. Additionally, a longer carbon chain size results in a lower value of ${ln\gamma }_{i}^{\infty },$ indicating a higher carotenoid-carboxylic acid affinity. In other words, solvents with non-polar characteristics have a favorable capacity to solubilize carotenoids (Fig. 3C).

COSMO-based models are valuable tools used in biological/chemical processes for predicting thermodynamic properties, thereby diminishing the need for extensive experimental work. Recently, some researchers have used COSMO-based models to predict the affinity of different carotenoids with several solvents. Yara-Varón et al. (2016) employed the COSMO-RS to determine the most effective green solvents for replacing n-hexane in the extraction of carotenoids from carrots. Through the analysis of σ-profile and solubility prediction, the researchers described that non-polar or weakly polar solvents are more suitable for extracting β-carotene, α-carotene, and lycopene. However, more polar solvents would be preferable if lutein was the target compound (Yara-Varón et al. 2016). Rajabi et al. (2017) also used the COSMO-RS to describe the relationship between β-carotene and some IL for its extraction from an organic phase composed of hexane. In this study, several cations and anions were evaluated. Ammonium-based ILs exhibited the highest capacity for extracting the target carotenoid from hexane. However, the selectivity of the ILs decreased as the alkyl chain length on the ammonium cation increased. In other words, the longer the alkyl chain, the stronger the interaction of the IL with n-hexane compared to β-carotene.

The COSMO-RS model was also applied to screen the performance of 24 distinct hydrophilic and hydrophobic DESs in the extraction of fucoxanthin from the *Tisochrysis lutea* algae. The carbon chain length of the carboxylic acids, used as HBD, was found to have a direct impact on the DES extraction capacity. Longer carbon chains enhanced the fucoxanthin extraction when thymol and tetrabutylammonium chloride (TBAC) were used as the HBA, while the opposite effect was observed when menthol was used as HBA. Among the five HBA evaluated, thymol had the best capacity for fucoxanthin obtaining (Xu et al. 2022), which aligns with the σ-profile of the compounds. Fucoxanthin molecule is predominantly distributed in the non-polar region, but also exhibits peaks in the HBD and HBA regions, with the HBA capacity being more pronounced due to the presence of ketone, ester, and epoxide functionalities. Hence, the ideal solvents should primarily be distributed in the non-polar region while also exhibiting some HBD character. Note that this consideration is crucial for the selection of DES components (Xu et al. 2022). Viñas-Ospino et al. (2023b) also employed the COSMO model to screen 68 hydrophobic and hydrophilic DES for extracting carotenoids from orange peels. β-carotene and β-cryptoxanthin were used as molecular probes due to their different polarities. The hydrophilic DES, owing to their high viscosity, were calculated to contain 30 wt.% water in the mixture. The results of ${ln\gamma }_{i}^{\infty }$ calculations revealed that strong-hydrophilic DES composed of choline chloride, as HBA, and urea, sorbitol, and sucrose as HBD were unable to solubilize both carotenoids, while the hydrophobic DES, with menthol as HBA and eucalyptol and camphor as HBD, exhibited the lower ${ln\gamma }_{i}^{\infty }$ values for β-carotene and β-cryptoxanthin. The researchers validated the model, and the experimental outcomes were consistent with the predicted results.

The COSMO-SAC model was utilized to investigate the extraction of β-carotene and astaxanthin from *Phaffia rhodozyma* biomass in cholinium-based IL aqueous solutions (Mussagy et al. 2022a). Various carboxylic acids were screened as anions, with different alkyl chain lengths, to determine the impact of the alkyl chain length on extraction capacity. It was observed that the longer the alkyl chain length, the greater the extraction capacity, as the anion is responsible for imparting the non-polar region to these cholinium-based ILs.

Despite the limited number of studies available in the literature, the COSMO model has demonstrated promising results in exploring the screening of tailor-made solvents for carotenoid recovery (Rajabi et al. 2017; Mussagy et al. 2022a; Xu et al. 2022; Viñas-Ospino et al. 2023b). Predictive tools such as the COSMO model are highly relevant in contributing to the viability of separation processes for valuable products like carotenoids obtained from microbial sources. Furthermore, the use of tailor-made solvents for microbial carotenoid extraction has also shown promising results, as discussed in the following sections.

## Microbial carotenoids extraction using ILs and DES

Through a straightforward literature analysis, it is evident that ILs have demonstrated effectiveness in extracting a wide range of compounds, including carotenoids from microorganisms (see Table 1). ILs function as cell disruption agents, enhancing the permeation and modification of microbial biomass cell walls. This facilitates the release of intracellular compounds, highlighting their potential to improve extraction efficiency (Torres-Valenzuela et al. 2020). In certain situations, incorporating a co-solvent, such as water or alcohols, can be advantageous in decreasing the viscosity of the IL. This decrease in viscosity enhances fluidity and promotes more efficient mass transfer, which may lead to quicker extraction and higher yields. However, it is essential to recognize that the addition of the co-solvent can influence the auto-partitioning behavior between the IL and the immiscible layer by altering the polarity and solubility. These changes have a significant impact on the selectivity and efficiency of the extraction process, ultimately determining how carotenoids are distributed within the system (Yu et al. 2022).

**Table 1:** Microbial carotenoids extraction using ionic liquids (ILs)

Microorganism	Type of biomass	ILs	Extraction conditions	Carotenoid recovered and yield	References
*Rhodotorula glutinis* CCT-2186	Wet cells	[DEAPA][Hex]	Homogenized using a rotatory orbital sample shaker, 90% (v/v) PIL concentration, 65 °C, 0.2 g/mL of cells for 1 h at 30 rpm	206.65 ± 10.75 μg/mL β-carotene, 112.82 ± 6.09 μg/mL torularhodin, 17.21 ± 1.99 μg/mL torulene	(Mussagy et al. 2019b)
*Phaffia rhodozyma NRRL* Y-17268	Wet cells	[Ch][But]	Homogenized using a magnetic stirrer hot plate mixer for 1 h at 65 °C and 300 rpm using 0.2 g/mL of cells	14.7 ± 1.8 (% w/w) astaxanthin and 21.2 ± 0.1 (% w/w) β-carotene	(Mussagy et al. 2022c)
*Haematococcus pluvialis*	Dried cells	[EMIM] [DBP]	10 mg biomass with 2.1 mL IL, 40% w/w IL at 45 °C for 90 min	Astaxanthin (≥ 70%)	(Desai et al. 2016)
*Haematococcus pluvialis* NIES-144	Wet biomass	[Emim] EtSO_4_	Cells from 1 mL of culture, 0.5 mL IL, vortex for 1 min at 28 °C	19.5 pg of astaxanthin per cell	(Praveenkumar et al. 2015)
*Haematococcus pluvialis* NIES-144	Freeze dried cells	[Emim] HSO_4_), [Emim] CH_3_SO_3_), [Emim] (CF_3_SO_2_)_2_N	200 mg cells, 6 mL of 6.7% (v/v) ILs in water, 30 °C, 500 rpm and 1 h	Astaxanthin (> 99%)	(Choi et al. 2019)
*Haematococcus pluvialis*	Powder	EAC	PILs-MALSE, 50 mg biomass, 5.2 mol/L EAC; 210 W microwave irradiation power for 50 s, 10 g/g liquid–solid ratio, 2 extractions	Astaxanthin (97.2%)	(Fan et al. 2019)
*Chaetoceros calcitrans* UPMC-A0010	Lyophilized biomass	DACARB	25 mg biomass, 3 mL of 90% (v/v) of IL concentration, 3 min and 25 °C	17.51 mg/g fucoxanthin	(Khoo et al. 2021b)

*[Ch][But]* choline butanoate, *DACARB* dialkylammonium diallylcarbamate, *[DEAPA][Hex]* 3-diethylamino-propylammonium hexanoate, *EAC* ethanolammonium caproate, *[Emim] (CF*_*3*_*SO*_*2*_*)*_*2*_*N* 1-ethyl-3-methylimidazolium bis(trifluoromethylsulfonyl)imide, *[Emim] CH*_*3*_*SO*_3_ 1-ethyl-3-methylimidazolium methanesulfonate, *[Emim] [DBP]* 1-ethyl-3-methylimidazolium dibutylphosphate, *[Emim] EtSO*_*4*_ 1-ethyl-3-methylimidazolium ethylsulfate, *[Emim] HSO*_*4*_ 1-ethyl-3-methylimidazolium hydrogen sulfate, *PILs-MALSE* protic ionic liquids-based microwave-assisted liquid–solid extraction

Mussagy et al. (2019b) evaluated the potential of 12 aqueous solutions of ammonium based protic ILs (PILs) for the permeabilization of *Rhodotorula glutinis* CCT-2186 cells and carotenoids extraction. The study found that the extraction of carotenoids was facilitated with by increased temperature, hydrophobicity, and concentration of PILs. Notably, the hexanoate-based ILs demonstrated the ability to recover up to fourfold carotenoids at 25 °C and sixfold at 65 °C compared to DMSO.

Fan et al. (2019) explored the use of PILs in combination with microwave-assisted solid–liquid extraction to recover microbial astaxanthin from *Haematococcus pluvialis*. The study evaluated various processing parameters, including PILs concentration, number of extractions, microwave irradiation power, microwave irradiation time, and solid–liquid ratio. The highest astaxanthin recovery (97%) was achieved using ethanolammonium caproate (EAC). This PIL exhibited a strong capacity to dissolve mannan, a primary component of the cell walls in *Haematococcus pluvialis*, thus, eliminating the need for a pre-treatment step to disrupt the cell walls prior to extraction, resulting in a significantly accelerated extraction process with a duration as short as 50 s.

The use of ionic liquids (ILs) as highly efficient and biocompatible alternatives for natural carotenoid extraction has shown significant potential, despite limited studies on their application in extracting carotenoids from microbial sources. Therefore, further research is essential to establish the complete efficacy of ILs in microbial carotenoid extraction. In these studies, it is imperative to prioritize ILs with eco-friendly properties, including low environmental impact and toxicity levels, high biodegradability, and accessibility from renewable sources. Given the numerous combinations of cations and anions available, a focused emphasis on tailor-made ILs with these environmentally favorable characteristics will be critical in advancing the environmental sustainability of microbial carotenoid extraction methodologies.

Incorporating physical properties similar to ILs, tailor-made DES offer several benefits, including reduced economic and environmental impacts, simpler synthesis routes, and decreased toxicity (Yu et al. 2022). In some cases, when natural compounds are used in the formulation of DES, they are referred to as natural deep eutectic solvents (NADES). Both DES and NADES are appealing alternatives to conventional VOCs due to their easy purification and potential (re)use in subsequent materials manufacturing. Nevertheless, these solvents exhibit high viscosities, which can pose challenges for mass transfer and limit the efficiency of extraction process (Gullón et al. 2020; Yu et al. 2022; Viñas-Ospino et al. 2023a). As listed in Table 2, some relevant studies in the literature have reported extraction from microbial sources.

**Table 2:** Microbial carotenoids extraction conditions using deep eutectic solvents (DES)

Microorganism	Type of biomass	DES	Extraction conditions	Carotenoid recovered and yield	References
*Phaffia rhodozyma* NRRL Y-17268	Wet cells	Choline chloride + Butyric acid, molar ratio = 1:2	Homogenized using a magnetic stirrer hot plate mixer for 1 h at 65 °C and 300 rpm using 0.2 g/mL of cells	47.9 ± 0.8 (% w/w) astaxanthin and 46.0 ± 1.0 (%w/w)] β-carotene	(Mussagy et al. 2022c)
*Rhodotorula mucilaginosa* CCT3892	Dried cells	Choline chloride + glycerol, molar ratio = 1:2	0.2 g biomass, 2 mL of DES with ethanol as adjuvant (30% v.v^−1^) incubation in a thermal bath at 50 °C and 1 h	0.110 ± 0.005 mg/mL of total carotenoids	(da Costa et al. 2020)
*Thalassiosira andamanica* CSIRCSMCRI 002	Frozen fresh biomass (70% moisture)	Benzyltriethyl ammonium chloride: Butane 1,4 diol (BTEA: BUT), molar ratio = 1:2	Room temperature, 40 min, and 3:1 solvent to biomass ratio	19.93 mg/g biomass Fucoxanthin	(Singh et al. 2022)
*Nannochloropsis oculata*	Dried biomass	Betaine: 1,2-propanediol (BP), molar ratio = 2:5	UAE, BP with the addition of water to the final 12.5%, 0.4 mL/40 mg biomass and dilution in 1 mL water; 40 °C for 40 min	5.53 mg astaxanthin equivalent/g DW (which 2.34 mg/g DW was violaxanthin)	(Gkioni et al. 2022)
*Haematococcus pluvialis* HP5	Freeze-dried biomass	Thymol: oleic acid, molar ratio = 3:1	5 wt% biomass/ solvent ratio, 6 h, room temperature	Astaxanthin (84.9 ± 3.7%)	(Pitacco et al. 2022)
*Scenedesmus* sp*.*	Powder	Fenchyl alcohol/thymol, molar ratio = 1:1	0.02:1 mass/solvent ratio, 60 °C for 70 min	6.26 ± 0.40 mg Lutein/g biomass	(Fan et al. 2022)

Mussagy et al. (2022c) used an aqueous solution (at 80% w/w) of choline chloride:butyric acid (molar ratio = 1:2) to increase the recovery of astaxanthin and β-carotene from *P. rhodozyma* NRRL Y-17268 by approximately 10% compared to the control (DMSO). The same researchers observed that the use of chloride:butyric acid (1:2 molar ratio) favored the extraction of astaxanthin, while the extraction of β-carotene was favored by using chloride:lactic acid (1:1 and 1:2 molar ratios).

Fan et al. (2022) investigated the potential of NADES in the recovery of lutein from *Scenedesmus* sp. The researchers found that lutein can be effectively extracted from microalgae using fenchyl alcohol:thymol (at equimolar ratio) at 60 °C for 70 min. The NADES enhanced the stability of lutein under high temperatures, exposure to light, and long storage periods. The study also revealed that the hydrogen bonding and van der Waals interactions played critical roles during target processing, clarified through theoretical calculations and nuclear magnetic resonance (NMR) analysis.

As depicted in this section, the utilization of DES and NADES in the extraction of microbial carotenoids remains relatively restricted. Similar to ILs, additional research is necessary to enhance the recovery and (re)usability of DES or NADES, with the ultimate goal of improving environmental and economic sustainability in industrial extraction processes. Given that the efficiency of the process relies on the composition of various biomass types, carotenoids, and tailor-made solvents precursors characteristics, further studies are required to identify optimal conditions and understand the interactions between DES and diverse matrices, thereby facilitating the scale-up of the process. Upon the extraction of microbial intracellular carotenoids, these carotenoids remain solubilized in the solvent. The following critical step involves the recovery of the carotenoids from the solvent for their final application. Although both ILs and DES exhibit high efficiency in extracting carotenoids from microbial biomass, the primary challenge lies in separating these target compounds from the final extract and efficiently recovering these tailor-made solvents for reuse, as detailed the subsequent section.

## Carotenoids separation from IL and DES extracts

**Fig. 4:**
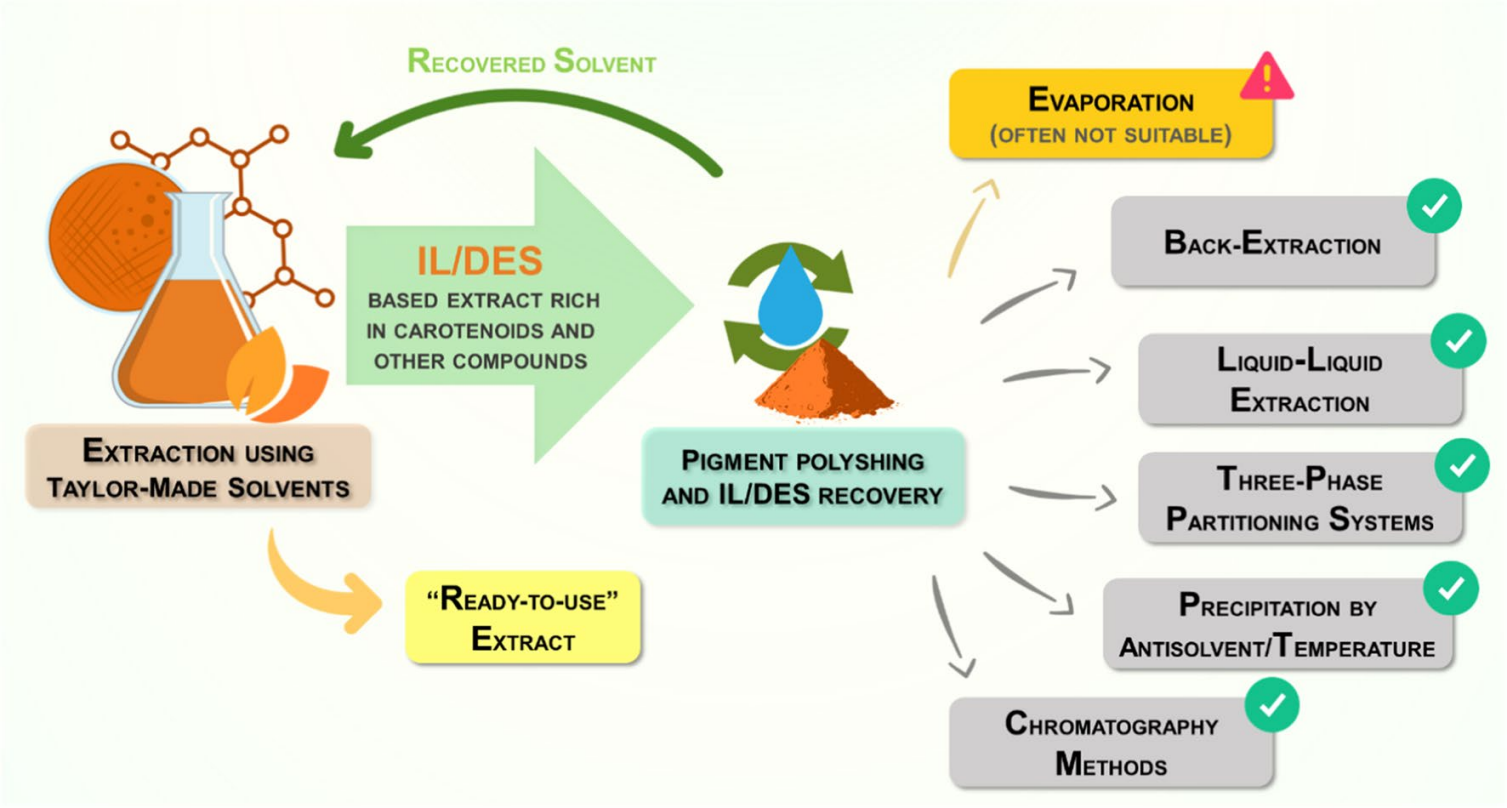
Main strategies for solvent recycling and polishing of carotenoids extracted using tailor-made solvents (ILs and DES)

The separation of carotenoids from tailor-made solvents is complicated by their lipophilic nature, which can result in the simultaneous extraction of other microbial compounds, such as proteins and lipids. This can pose challenges depending on the intended use of the extracted compounds (Mussagy et al. 2021). Furthermore, the low vapor pressure of ILs and DES presents a significant challenge, particularly considering the thermolabile nature of the recovered intracellular carotenoids (Benvenutti et al. 2019). Therefore, traditional evaporation methods are often impractical for their recovery, necessitating alternative strategies. Some of the significant approaches that are commonly used to address the challenges associated with solvent recovery for carotenoid-rich extracts include liquid–liquid extraction (LLE), back-extractions, three-phase partitioning, precipitation induced by the addition of anti-solvents or temperature, and chromatographic methods (Fig. 4).

In contrast to well-defined purification methods of carotenoids from fruits and vegetable by-products, studies involving microbial biomass often lack detailed information on the carotenoid isolation step. Our focus herein was extended beyond the purification of extracts from microbial biomass since purification steps typically remain consistent regardless of the extract’s source. Mussagy et al. (2019b) developed a three-phase partitioning system using 3-diethylaminopropylamine hexanoate protic IL (PIL) and tripotassium phosphate (K_3_PO_4_). This system enabled the simultaneous extraction of β-carotene, torularhodin, and torulene from *R. glutinis* yeast biomass while allowing the solvent to be recycled for at least three more extraction cycles. The process involved creating a three-phase partitioning (TPP) unit (liquid–solid-liquid) by adding a concentrated aqueous solution of K_3_PO_4_ to a carotenoid-rich PIL solution. This resulted in the precipitation of most carotenoids as a solid fraction at the interface between the top PIL-rich liquid phase and the bottom K_3_PO_4_-rich liquid phase. Similarly, Suarez Ruiz et al., (2018) used a TPP system composed of polyethylene 400 (PEG400) and cholinium dihydrogen phosphate to fractionate lutein from proteins in the *Neochloris oleoabundan (N. oleoabundans)* microalgae biomass. Subsequently, the same researchers proposed a back-extraction technique that utilized aqueous biphasic systems (ABS) for the purpose of microalgae biorefinery (Suarez Ruiz et al. 2020).

Silva et al., (2023) proposed an LLE-based back-extraction method for the recovery of β-carotene and astaxanthin extracts from *P. rhodozyma* biomass in ammonium-based PILs. Following the successful application of propylammonium octanoate ethanolic solutions in pigment extraction using SLE, ethyl acetate (EtOAc) was used as an antisolvent to induce two-phase partitioning system. This led to the formation of a carotenoid-EtOAc rich (top) phase and a PIL-rich (bottom) phase. The top phase was then evaporated for solvent recycling and pigment recovery from EtOAc. Ueda et al. (2023) utilized a similar approach to isolate β-carotene and lycopene from an ethanolic cholinium-based IL extract derived from *Eugenia Uniflora* pulp.

Antisolvents can also be applied to induce pigment precipitation, as demonstrated by Gao et al., (2020a, b) in the isolation of astaxanthin from shrimp waste applying phosphonium and ammonium based ILs. Given the hydrophobic nature of carotenoids, water is the most common antisolvent, as demonstrated by De Souza Mesquita et al. (2020). These researchers had also formerly proposed a different strategy for the purification of *B. gasipaes* fruit extract, where IL was precipitated at − 80 °C and the carotenoids-rich ethanolic extract was subsequently evaporated under low pressure (De Souza Mesquita et al. 2019). This thermal precipitation strategy was first proposed by Martins and Rosso (2016), achieving the recovery of lycopene from tomato samples and solvent recycling by precipitating the imidazolium-based ILs at -40 °C. Despite the potential use of precipitation or anti-solvents for separation of carotenoids and the extractant solvents, there are instances where these methods are not effective. In a study conducted by Murador et al.,(2019) efforts isolate carotenoids from an extract rich in carotenoids obtained from orange peel using 1-n-butyl-3-methylimidazolium chloride precipitation at temperatures of -40 °C/-80 °C were unsuccessful. This outcome is likely due to the predominance of xanthophylls in orange peels, which may hinder the precipitation of ILs owing to the presence of hydroxyl groups. In contrast, tomatoes and *B. gasipaes* fruits are rich in carotenes, which may be more readily precipitated by ILs. These findings highlight the importance of considering the type of carotenoids present in the natural matrices when selecting a separation method.

Alternatively, chromatographic methods utilizing two distinct chromatography columns (i.e., XAD-7HP and XDA-6 resins) were successfully applied for the separation of ILs and the desired carotenoids. However, chromatography posed limitations for its application on the food industry due to major drawbacks such as the need of additional solvents, resin consumption, low throughput, and high equipment costs. These constrains led Khoo et al., (2021a) to propose a different approach where CO_2_-based alkyl carbamate ILs were utilized to recover lutein from *C. sorokiniana* microalgae. The low distillation points of these solvents allow the ILs to be separated from the extracted compounds by a simple evaporation procedure.

According to DES, two purification methods based on hydrophobic switchable NADES have been proposed by Stupar et al., (2021) and Zhang et al., (2023) for recovering β-carotene from pumpkin and millet, respectively. In these approaches, pigments were precipitated by switching the polarity of the NADES to the hydrophilic form, in a simple and fast procedure. Stupar et al., (2021) used ammonium hydroxide to react with the C8:C10 fatty acid-based NADES, which resulted in a hydrophilic phase, and the samples were then subjected to centrifugation to separate the precipitated carotenoids from the liquid phase. Conversely, Zhang et al., (2023) took an additional step by recycling and reusing the switchable DES in five extraction cycles. A HCl solution was added to the N,N-dimethylcyclohexylamine and n-butanol-based NADES, leading to the formation of a hydrophilic solution. Thereafter, β-carotene solidified on the wall of the tube, facilitating its collection. Next, a NaOH solution was added to the hydrophilic DES, and the resulting hydrophobic DES was recycled to extract new samples.

The disparity in the number of studies proposing a purification step for ILs as compared to DES is evident, highlighting a significant gap in the literature for the latter, especially regarding carotenoid recovery. This discrepancy can be attributed to several factors. Firstly, the extensive exploration and adoption of ILs in various applications due to their well-established properties and versatility have led to a great number of studies on their purification strategies for different compounds, including carotenoids. On the other hand, being a relatively “newer” class of solvents, DES may not have received the same level of attention and thorough exploration, leading to a limited understanding of their properties and interactions with carotenoids. Additionally, it is noteworthy that DES are generally considered more biocompatible than ILs, prompting several studies to propose the direct use of DES extracts in “ready-to-use” form, thereby bypassing the need for extensive purification steps (Rente et al. 2022). This emphasis on the biocompatibility of DES may further explain the limited focus on purification strategies for these solvents in the current literature. Table 3 offers a comprehensive overview, outlining key advantages and drawbacks of employing these solvents for carotenoid purification.

**Table 3:** Comparative analysis of ILs and DES for carotenoid polishing and solvent recovery

	Ionic liquids (ILs)	Deep eutectic solvents (DES)
Strengths	▪ Broad solubility range; effective for various compounds, including carotenoids	
	▪ Can be tailored for specific applications	
	-	▪ Generally considered more environmentally friendly and biocompatible, often making it possible to directly apply the extract without need for further purification steps
	-	▪ Often considered more cost-effective due to simpler synthesis
Drawbacks	▪ High viscosity may impede mass transfer; can hinder extraction efficiency	
	▪ Low vapor pressure complicates solvent recovery; may require energy-intensive processes	
	▪ Some ILs may have environmental concerns due to toxicity and persistence	-
	▪ Synthesis and purification processes may contribute to higher costs	-

Furthermore, a potential alternative to the aforementioned techniques for recycling and reusing neoteric solvents is the incorporation of these extracts containing solvents directly into the final formulation, resulting in the development of novel functional materials/products. Mussagy et al. (2022c) successfully employed this approach to produce biodegradable active biofilms from corn starch using the [Ch]Cl:But (1:1) extract, which is rich in carotenoids, as a plasticizer agent. Importantly, the integration of the extraction and formulation processes did not negatively impact the biological properties of the carotenoids, indicating that certain solvents can be directly utilized in the formulation of new products without the need for separation or polishing of the carotenoids from the solvents.

Recent studies have highlighted the potential of ionic liquids (ILs) and deep eutectic solvents (DES) as eco-friendly alternatives to traditional volatile organic solvents for the extraction of microbial carotenoids. However, the environmental impact of these novel solvents needs to be assessed to ensure that their use does not result in negative environmental consequences that may offset their benefits. Unfortunately, there is a lack of published literature on the application of Life Cycle Assessment (LCA) to the extraction of carotenoids using ILs and DES, or even for other classes of biomolecules. The use of LCA is critical for decision-making in the selection, design, and optimization of sustainable technologies (Cassani et al. 2022). One exception is the work of Martins et al. (2021), who conducted an environmental analysis of a proposed process for the extraction and fractionation of chlorophyll and fucoxanthin from *Saccharina latissimi* using ILs. The authors reported that the main contributors to the environmental impact were fossil resource scarcity and electricity consumption, and the reuse of ILs resulted in a reduction of 8–14% of the environmental impact. Further research is needed to compare the extraction of carotenoids with ILs and DES to other methods reported in the literature, such as the comparison made by Kyriakopoulou et al. (2015) between conventional solvent extraction and innovative green extraction methods using process intensification strategies with microwaves and ultrasounds.

It is recommended that a LCA be conducted in conjunction with a techno-economic analysis (TEA) for optimal evaluation of the economic viability of a technology or process. TEA provides a comprehensive analysis of the costs and benefits associated with a particular technology or process, including cost–benefit analysis, risk assessment, comparison with alternatives, scale-up considerations, and resource allocation (Jin et al. 2018). By conducting a TEA, it is possible to quantify and understand the costs associated with the production and use of these solvents in comparison to conventional solvents. Martins et al. (2021) demonstrated that when recycling scenarios were included in the economic analysis, production costs decreased. Additionally, the authors found that lower processing costs can be achieved by recycling only 20% of IL in the final process proposed.

In summary, the selection of tailor-made solvents is influenced by various factors such as solubility, tailorability, environmental impact, and cost. The strategic choice of solvent precursors depends on the specific demands of the extraction process and the desired outcomes. As the field advances, continuous research and optimization are necessary to realize the full potential of these tailor-made solvents in achieving effective processing platforms where microbial carotenoid polishing and solvent recovery are also guaranteed. This approach is crucial in addressing challenges and maximizing the benefits these solvents offer.

## Final remarks

The use of tailor-made solvents, including ILs and DES, has garnered increasing attention in recent literature, particularly in the context of bioprocesses. These new solvents find diverse applications in the solid–liquid recovery of various biomolecules, with a specific focus on microbial carotenoids, due to their unique properties such as broad solubility range and biocompatibility. For screening new tailor-made solvents for the recovery of microbial carotenoids, in silico approaches such as COSMO-RS or SAC may be employed in the future to streamline experimental solvent screening. This can aid in identifying the most suitable ILs and DES for a specific carotenoid solubilization and reduce the cost of processes, especially in time-consuming experimental studies involving many combinations of tailor-made solvents precursors. Despite these advancements, conventional VOCs continue to be the preferred for microbial carotenoid extraction, driven by their long-standing familiarity and practicality, despite associated environmental concerns. Alternative eco-friendly solvents, such as biosolvents like ethyl acetate or supercritical solvents like supercritical CO_2_, have emerged in reported studies, offering high intracellular carotenoid extraction and recyclability. While the use of ILs and DES for microbial carotenoid extraction remains driven by scientific curiosity in academia, the potential economic feasibility of these solvents is yet to be demonstrated in practical applications.

As experts in carotenoid production and recovery using microorganisms (i.e., yeasts and bacteria), we emphasize the importance for the scientific community involved in microbial carotenoid extraction to carefully consider the biocompatibility of these solvents. Many anions/cations or HBD/HBA may not be biocompatible, and therefore, solvent biocompatibility tests should be conducted before subjecting them to any bioprocess. We also draw attention to the commercial cost of these tailor-made solvents compared to conventional biosolvents. It is necessary to question *whether the extraction efficiency of these solvents justifies their cost in the final product.* Technical–economic analysis can provide insight into these concerns. With the regard to the environment, when new solvents are synthesized and labeled as “*green”*, it is crucial to assess their environmental impact throughout the entire process, from the production of raw precursors to the synthesis of the tailor-made solvents, and finally, to the obtainment of the target product. Conducting a LCA of the entire process facilitate informed and assertive decision-making.

Anyway, considering their advantages, particularly in the selective recovery of target carotenoids, and following a comprehensive evaluation of life cycle assessment, biocompatibility, and economic analysis, we believe that these solvents have the potential to serve as viable alternatives to conventional methods, thereby paving the way for a more sustainable and efficient future in microbial carotenoid extraction.

## Data Availability

All data generated or analyzed during this study are included in the submitted manuscript.
